# Lipidomics Revealed Alteration of Sphingolipid Metabolism During the Reparative Phase After Myocardial Infarction Injury

**DOI:** 10.3389/fphys.2021.663480

**Published:** 2021-03-12

**Authors:** Tong Hua, Qiankun Bao, Xue He, Wenbin Cai, Jinlong He

**Affiliations:** ^1^Tianjin Key Laboratory of Metabolic Diseases, Collaborative Innovation Center of Tianjin for Medical Epigenetics, Department of Physiology and Pathophysiology, Tianjin Medical University, Tianjin, China; ^2^Tianjin Key Laboratory of Ionic-Molecular Function of Cardiovascular Disease, Department of Cardiology, Tianjin Institute of Cardiology, The Second Hospital of Tianjin Medical University, Tianjin, China

**Keywords:** lipidomics, sphingolipid, myocardial infarction, ceramide kinase(Cerk), ceramide-1-phosphate(C1P)

## Abstract

Aberrant sphingolipid metabolism contributes to cardiac pathophysiology. Emerging evidence found that an increased level of ceramide during the inflammatory phase of post-myocardial infarction (MI) served as a biomarker and was associated with cardiac dysfunction. However, the alternation of the sphingolipid profile during the reparative phase after MI is still not fully understood. Using a mouse model of the left anterior descending ligation that leads to MI, we performed metabolomics studies to assess the alternations of both plasma and myocardial sphingolipid profiles during the reparative phase post-MI. A total number of 193 sphingolipid metabolites were detected. Myocardial sphingolipids but not plasma sphingolipids showed marked change after MI injury. Ceramide-1-phosphates, which were accumulated after MI, contributed highly to the difference in sphingolipid profiles between groups. Consistently, the expression of ceramide kinase, which phosphorylates ceramides to generate ceramide-1-phosphates, was upregulated in heart tissue after MI injury. Our findings revealed the altering sphingolipid metabolism during the reparative phase post-MI and highlighted the potential role of ceramide kinase/ceramide-1-phosphate in ischemic heart disease.

## Introduction

Cardiovascular diseases were the most common underlying cause of death in the world, accounting for an estimated 31.5% of all global deaths (Benjamin et al., 2019). Heart failure (HF) after myocardial infarction (MI) is the major driver of late morbidity, mortality, and healthcare cost (Cahill and Kharbanda, 2017). Since both the incidence and prevalence of post-MI leading to HF have continually climbed (Benjamin et al., 2019), it is important to identify treatable conditions potentially contributive to HF progression.

Emerging evidence suggest that aberrant sphingolipid metabolism and signaling play a crucial role in cardiac pathophysiology (Kovilakath and Cowart, 2020; Kovilakath et al., 2020). Sphingolipids, including ceramide (Cer), ceramide-1-phosphate (Cer1P), sphingomyelins (SM), glycosphingolipids, and so on, are defined by their sphingoid base, which is generated by condensation of an amino acid with an acyl-CoA (Kovilakath and Cowart, 2020). Sphingolipids, not only act as the essential components of the eukaryotic cell membrane, but also serve as signaling molecules to regulate many cellular processes including cell survival, proliferation, apoptosis, and protein synthesis (Spiegel et al., 1998; Hannun and Obeid, 2018). For example, studies demonstrated that increased Cer levels in mammalian heart tissues during acute MI (AMI) were associated with higher cell death rates in the left ventricle and deteriorated cardiac function (Yu et al., 2015; Hadas et al., 2020). Hence, increased attention has been focused on perturbed sphingolipid metabolism associated with cardiovascular diseases (Laaksonen et al., 2016; Parveen et al., 2019), highlighting the importance to study the alteration of sphingolipid profile in a certain condition and the underlying mechanism.

Cardiac remodeling is a dynamic and time-dependent process, with changes occurring in both the infarcted and non-infarcted regions of the ventricle (Olivetti et al., 1990; Yang et al., 2002). There are two well-defined phases of healing after MI in the mouse model. The inflammatory phase (peak day ∼3–4 post-MI) is associated with acute inflammation with intense cellular infiltration. The reparative phase (peak day ∼7 post-MI) is the following resolution and repair period with active resolution of inflammation, quiescence of cell activity, and scar stabilization and maturation over 14 days in mice (Prabhu and Frangogiannis, 2016). Cer, as the key metabolite of sphingolipids, has been reported to be highly related to MI. Plasma Cer was found markedly increased at day 1 and day 2 after AMI in patients (de Carvalho et al., 2018). Similarly, myocardial Cer concentration was increased at 4 h post-MI in rats compared to sham groups (de Carvalho et al., 2018). However, the sphingolipid metabolism of the heart during the reparative phase post-MI is still not fully understood.

In the present study, we investigated the metabolomic profile of plasma and myocardium of mice at the reparative phase after MI surgery using a liquid chromatography/mass spectrometry (LC-MS) based approach. Our results indicated that myocardial Cer1P derived from Cer was upregulated and contributed highly to the alteration of sphingolipid profile after MI injury. Accordingly, this change might attribute to the upregulation of Cerk expression in heart tissue post-MI.

## Materials and Methods

### Animal Myocardial Infarction Models

Study protocols involving the use of animals were approved by Institutional Animal Care and Use Committee of Tianjin Medical University. Eight-week-old C57BL/6 mice were purchased from Vital River Laboratory Animal Technology (Beijing). For all the experiments, male mice were used because of gender difference of lipidomic profile (Barrier et al., 2010). All mice were maintained under a 12:12 h light/dark cycle before and throughout experiments. MI surgery was performed as previously described (Ding et al., 2017). Briefly, mice were anesthetized in an induction chamber with 3% isoflurane mixed with 1 L/min of 100% O_2_, while the anesthesia during the surgery was maintained at 1.5% isoflurane. The mice were operated on a heating plate (36°C), then the left anterior descending branch of the coronary artery (LAD) was permanently ligated above branching using a 6.0 silk suture, about 1 mm below the tip of the left auricle. In sham surgery, only the chest and pericardium were opened but no LAD ligation was carried out. The mouse thoracic cavity was then closed and sutured.

### Echocardiography

Cardiac function was assessed by transthoracic echocardiography. Echocardiographic monitoring was performed at baseline (before surgery) and 7 days post-surgery before the tissue was harvested via using an ultrasound system with a linear transducer with 32–55 MHz frequency combined with Vevo 2100 software (VisualSonics Inc., Toronto, Canada). B-mode tracings in the long-axis views were recorded, and dimensions of systolic and diastolic myocardium were measured. Parameters are calculated according to the VisualSonics standard measurements and calculations.

### Triphenyl Tetrazolium Chloride Staining

Triphenyl tetrazolium chloride (TTC) staining was performed as previously reported (Ding et al., 2017). Briefly, mouse heart was dissected after infusion of 10% Alcian blue (Cat No. A3157, Sigma-Aldrich) and frozen at −20°C for 20 min, then cut into 1-mm-thick slices from apex to base. The slices were incubated in 1.5% TTC at 37°C for 15 min.

### LC-MS Method for Metabolomics

Lipids were extracted from plasma and tissue of mice by the methyl-tert-butyl ether (MTBE)-based method as described (Matyash et al., 2008; Yan et al., 2020) with minor modifications. Briefly, 20 μL plasma or 10 mg heart tissue spiked with internal standard mixture was blended into 400 μL 75% methanol. MTBE (1 mL) was added and vortexed for 1 h. Phase separation was induced by adding 250 μL Milli-Q water. After 10 min of incubation at room temperature and centrifugation at 14,000 g for another 10 min, the upper (organic) phase was collected and evaporated to dryness.

Targeted profiling of sphingolipids involved the use of a 5500 QTRAP hybrid triple quadrupole linear ion trap mass spectrometer (AB Sciex, Foster City, CA) equipped with a Turbo Ion Spray electrospray ionization source. The mass spectrometer was operated with Analyst software (version 1.6.1, AB Sciex, Foster City, CA). Lipid species were detected by multiple reaction monitoring (MRM) scanning mode. The dwell time used for all MRM experiments was 5 ms. The ion source parameters were CUR = 30 psi, GS1 = 30 psi, GS2 = 30 psi, CAD = MEDIUM, TEMP = 500°C, IS = 5500 V (for positive ions) or -4500 V (for negative ions). Chromatographic separation involved the use of a UPLC BEH C18 column (1.7 μm, 100 × 2.1 mm i.d.) consisting of ethylene-bridged hybrid particles (Waters, Milford, MA). The column was maintained at 25°C and the injection volume was set to 1 μL. Solvent A: 60% acetonitrile. Solvent B: acetonitrile/isopropanol (1/9, v/v). The mobile-phase flow rate was 0.25 mL/min. The gradient started from 25% solvent B and was maintained for 3 min, then 3–15 min solvent B to 99% and maintained for 2 min, and 17-19 min solvent B reduced to 25% and maintained for 1 min.

Multiquant software (version 3.0.2, AB Sciex, Foster City, CA) was used to process raw data. Metaboanalyst 3.0^1^ (Xia and Wishart, 2016) was used for data analysis and visualization, including partial least squares discriminant analysis (PLS-DA), the goal of fold change analysis, variable importance in projection (VIP) scores, and hierarchical clustering and presenting as a heatmap.

### Quantitative Real-Time Polymerase Chain Reaction

Total RNA was extracted from hearts of mice with QIAzol and purified by the use of the QIAGEN RNeasy Mini Kit (Cat No. 74104), then 1 μg of RNA was reverse transcribed with SuperScript III and random primers (Cat No. 12574035, Thermo Fisher, Grand Island, NY), as defined by the manufacturers’ manuals. Real-time PCR involved the Brilliant II SYBR Green qPCR Master Mix (Stratagene, CA, United States) and the StepOnePlus Real-Time PCR System (Applied Biosystems, Waltham, MA). Results were normalized to GAPDH. The following primer sequences for murine tissue were used (gene subsequent sequences of forward and reverse primers): GAPDH (5′-CAT GGC CTT CCG TGT TCC TA-3′, 5′-GCG GCA CGT CAG ATC CA′); Cerk (5′-GAC TGG GAG CAC TGA CAC AA-3′, 5′-GAG GAT GAG GGG AGG CCA TA-3′).

### Western Blotting

Lysates of the left ventricle were acquired by direct lysis in a 50 mM Tris buffer (pH 6.7) comprising a complete protease inhibitor cocktail (Cat No. 04693132001, Roche, Indianapolis, IN) and PMSF (Cat No. P0100, Solarbio Life Sciences, Beijing). The protein samples (20μg) were separated by SDS-PAGE, followed by western blotting as described previously (He et al., 2018). Primary antibody against Cerk (Cat No. sc-376730) was from Santa Cruz (Santa Cruz, CA). Primary antibody against GAPDH (Cat No. 60004-1-Ig) and secondary antibody against mouse (Cat No. SA00001-1) were from Proteintech (Rosemont, IL). Membranes were visualized by using an ECL Western blotting detection kit (Cat No. 34580, Thermo Fisher Scientific, Waltham, MA) in a ChemiScope3600 Mini chemiluminescence imaging system (Clinx Science Instruments, Shanghai). The bands were quantified using NIH Image J software.

### Immunofluorescence Staining

Seven μm frozen sections of heart were fixed in 4% paraformaldehyde for 15 min, permeabilized with 0.5% Triton X-100 in PBS for 30 min, blocked with 1% BSA for 30 min, and incubated with primary antibodies at 4°C overnight, then with Alexa fluor-conjugated secondary antibodies. Fluoroshield mounting medium with DAPI was used to cover slides. Images were visualized under an Olympus inverted microscope equipped with a charge-coupled camera.

### Statistical Analysis

All the data were presented as mean ± standard error of the mean (SEM). Statistical analysis was performed using GraphPad Prism software v7.0. The number of biological replicates and statistical significance are specified in figure legends. Differences between the two means were tested using unpaired two-tailed Student’s *t*-test. *P* < 0.05 was considered statistically significant.

## Results

### Sphingolipid Profile Altered in Mice After Myocardial Infarction Injury

**FIGURE 1:**
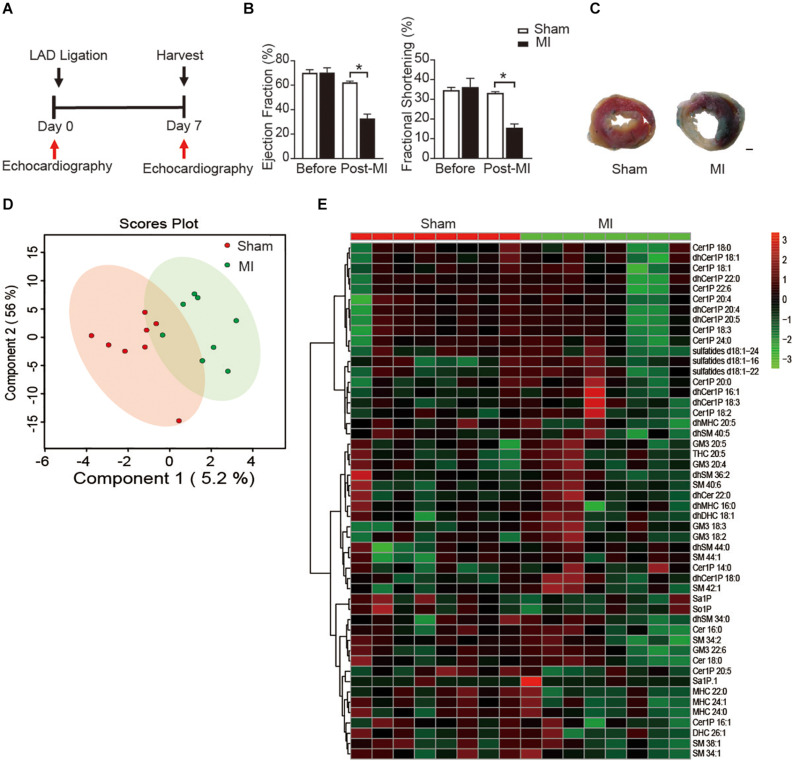
Plasma sphingolipid profile of mice at day 7 post-MI. **(A)** Timeline of the myocardial infarction (MI) experiment. **(B)** Quantification of ejection fraction (EF) and fraction shortening (FS). Data are mean ± SEM, *P < 0.05, *n* = 6. **(C)** Left ventricular (LV) tissue sections stained with Alcian blue and 2,3,5- triphenyltetrazolium chloride (TTC) at day 7 after MI. Scale bar, 500 μm. **(D)** PLS-DA score plot of the sphingolipid profile. **(E)** Heatmap showing the plasma sphingolipid profile of mice at day 7 post-MI. The colors represent increasing (red) or decreasing (green) expression, with the intensity reflecting the corresponding concentration.

To study the metabolic status of sphingolipids in the reparative phase of MI injury, we employed an MI model with ligating the left anterior descending (LAD) of mice. Cardiac function was measured with echocardiography at day 7 post-MI (Figure 1A). Both ejection fraction (EF) and fractional shortening (FS), the two main indicators to assess the systolic function of left ventricular (LV), were comparable between the sham group and the MI injury group before the surgery. Ligation of LAD induced a significant decrease of EF and FS at the 7 days after the surgery (Figure 1B), which is consistent with our previous reports (Ding et al., 2017; Fang et al., 2018). Also, the TTC staining of LV further confirmed the success of the MI model (Figure 1C).

Next, to get insight into the potential alteration in plasma sphingolipid profile in MI injury mice, LC-MS analyses with both positive ion mode and negative ion mode were performed. A total number of 193 metabolites were detected including Cer, dihydroceramide (dhCer), Cer1P, dihydroceramide-1-phosphate (dhCer1P), dihexosylceramide (DHC), dihydrodihexosylceramide (dhDHC), monohexosylceramide (MHC), dihydromonohexosylceramide (dhMHC), SM, dihydrosphingomyelin (dhSM), GM3 Ganglioside (GM3), sulfatide, sphingosine (So), sphingosine-1-phosphate (So1P), sphingasine (Sa), and sphingasine-1-phosphate (Sa1P). PLS-DA was used to identify patterns that categorized the two types of samples based on sham and MI. However, the 2-dimensional score plot of PLS-DA proved the classification model could not completely separate the samples by their groups (Figure 1D), suggesting the plasma sphingolipid profile might not be largely influenced during the reparative phage post-MI. Consistently, the relative concentrations of the top 50 metabolites shown in the heatmap (Figure 1E) were comparable between groups.

**FIGURE 2:**
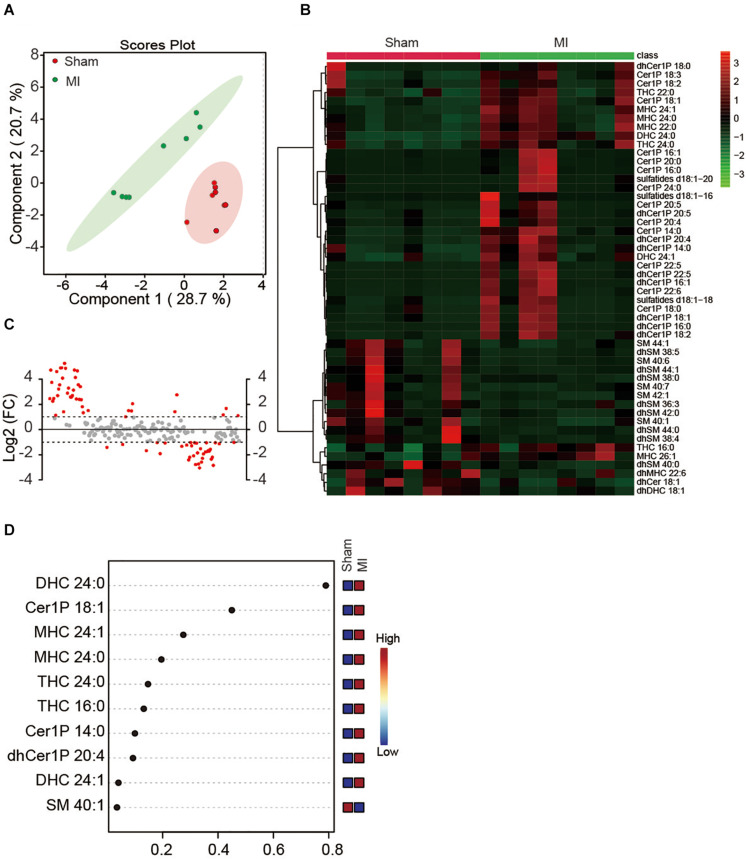
Myocardial sphingolipid profile of mice at day 7 post-MI. **(A)** The sparse PLS-DA score plot of the sphingolipid profile. **(B)** Heatmap showing the myocardial sphingolipid profile of mice at day7 post-MI. The colors represent increasing (red) or decreasing (green) expression, with the intensity reflecting the corresponding concentration. **(C)** The goal of fold change (FC) analysis of the absolute value of change between MI and Sham group means. Metabolites with Log2 (FC) ≥ or ≤ 1 were labeled red. **(D)** The top 10 most significant metabolites based on variable importance for prediction (VIP) scores from PLS-DA. The X-axis shows the correlation scores and the Y-axis the metabolites. Color bars show the median intensity of variables in the respective groups.

Since the plasma might dilute the metabolites and dismissed the potential difference between treatments, we next investigated whether MI injury induced a change of myocardial sphingolipids in heart tissue. Sphingolipids in LV were extracted and analyzed using the metabolomics method with LC-MS in both positive ion mode and negative ion mode. Similarly, a total number of 193 metabolites were detected including Cer, dhCer, Cer1P, dhCer1P and et al. In contrast to the results in plasma, the two-dimensional score plot of sparse PLS-DA proved the classification model could perfectly separate the samples of heart tissue by their groups (Figure 2A), indicating an obvious change of myocardial sphingolipids profile in LV. The concentrations of the top 50 changed metabolites were shown in Figure 2B. The magnitude of changes in sphingolipids between the MI and sham group were visualized by fold change (FC) analysis (Figure 2C). The threshold of FC was set to 2. Among the 42 upregulated metabolites, the top 5 were dhCer1P 16:0 (log2(FC) = 5.26), Cer1P 22:6 (log2(FC) = 4.90), Cer1P 22:5 (log2(FC) = 4.89), dhCer1P 16:1 (log2(FC) = 4.86) and dhCer1P 22:5 (log2(FC) = 4.72). A total of 34 metabolites, among which the top 5 were dhSM 38:5 (log2(FC) = −3.05), dhSM 44:0 (log2(FC) = −2.85), SM 385 (log2(FC) = −2.78), dhSM 38:4 (log2(FC) = −2.74) and SM 40:6 (log2(FC) = −2.59), were reduced after MI injury (Figure 2C). In addition, variable importance for prediction (VIP) scores were calculated, and the top 10 sphingolipids mostly contributing to the classification were shown on the Y-axis (Figure 2D). DHC 24:0, Cer1P 18:1, and MHC 24:1 were significantly increased in heart tissue after MI injury and contributed highly to the alteration of sphingolipid profile (Figure 2D). Taken together, we found myocardial but not plasma sphingolipid profile was markedly changed in mice at day 7 after MI injury via employing an unbiased LC-MS analysis.

### The Variations of the Sphingolipids After MI Injury

**FIGURE 3:**
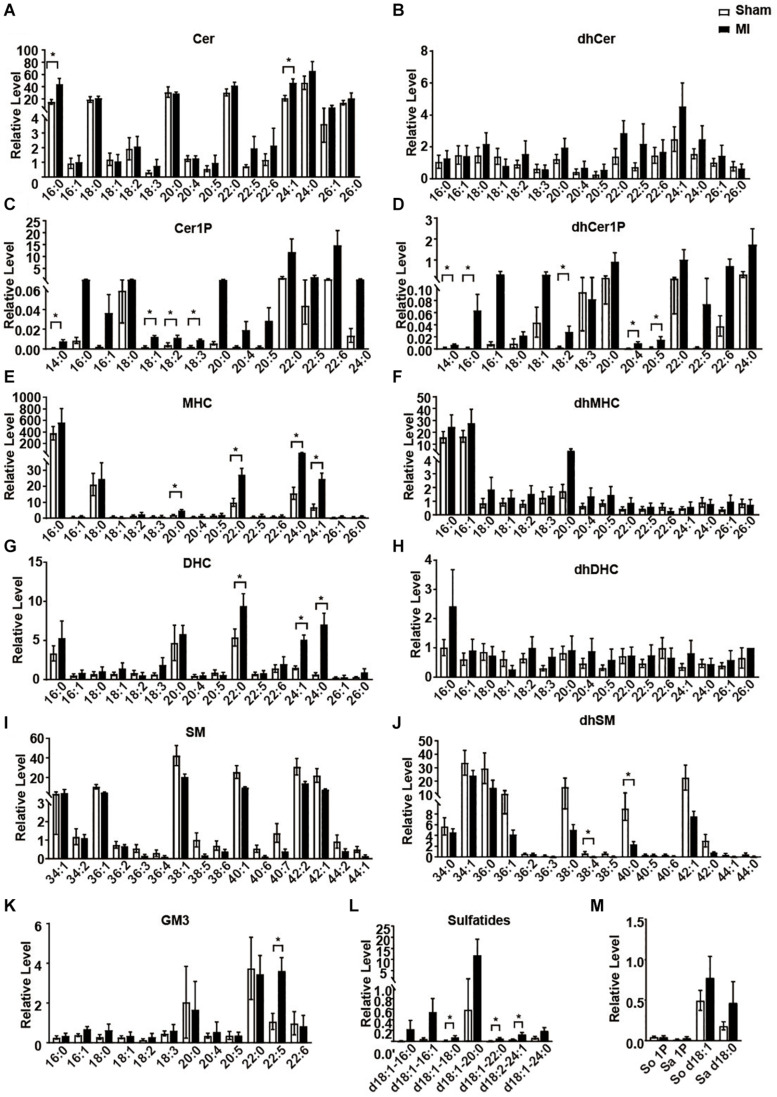
The relative levels of myocardial sphingolipids in the Sham and post-MI mice. **(A–M)** Sham or MI surgery was performed in mice. Liquid chromatography-tandem mass spectrometry detection of sphingolipids-derived metabolites in the heart tissue. Data are mean ± SEM, *n* = 8, *P < 0.05, unpaired two-tailed *t*-test.

We next statistically analyzed the relative level of each metabolite of sphingolipid. Consistent to previous studies (Laaksonen et al., 2016; de Carvalho et al., 2018), all the Cers showed an increasing trend in the MI group compared to the sham group (Figures 3A,B). However, only Cer 16:0 and Cer 24:1 showed a statistically significant increase (Figure 3A). As metabolites of Cer, all the Cer1P also showed a similar trend as Cer (Figures 3C,D). Cer1P 14:0, Cer1P 18:1, Cer1P 18:2, and Cer1P 18:3 were significantly upregulated in MI group (Figure 3C). Concentration of the dhCer1Ps including dhCer1P 14:0, dhCer1P 16:0, dhCer1P 18:2, dhCer1P 20:4, and dhCer1P 20:5 was increased with significance (Figure 3D). Levels of four MHC (MHC 20:0, MHC 22:0, MHC 24:0, and MHC 24:1), three DHC (DHC 22:0, DHC 24:1, and DHC 24:0), and one GM3 (GM3 22:5) were significantly higher, while the levels of two dhSM (dhSM 38:4 and dhSM 40:0) were significantly lower in MI groups (Figures 3E,G,J,K). Although sulfatide d18:1-18:0, sulfatide d18:1-22:0, and sulfatide d18:2-24:0 showed a relatively low level among all the sulfatides detected, they were significantly increased in the MI group (Figure 3L). Other metabolites including all the dhCer (Figure 3B); dhMHC (Figure 3F); dhDHC (Figure 3H); SM (Figure 3I); So, Sa, So1P, and Sa1P (Figure 3M) were not changed in the heart following MI injury.

### Expression of Cerk Was Upregulated in the Heart in Post-MI Mice

**FIGURE 4:**
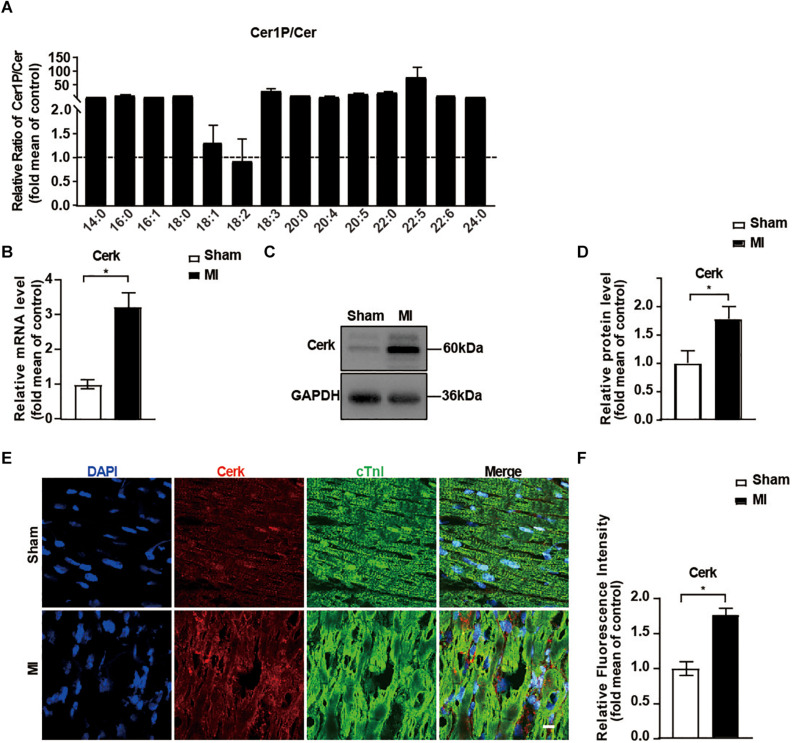
Expression of Cerk was upregulated in the myocardium after MI. **(A)** The relative ratios of Cer1P to Cer detected with LC-MS. Ratios of Cer1P to Cer in Sham group were set to 1. Data are mean ± SEM, *n* = 8. **(B)** RT-PCR analysis of mRNA level of Cerk in heart tissue. Data are mean ± SEM, *n* = 5, *P < 0.05, unpaired two-tailed *t*-test. **(C,D)** Western blot analysis and quantification of Cerk protein levels. Data are mean ± SEM, *n* = 5, *P < 0.05, unpaired two-tailed *t*-test. **(E,F)** Immunofluorescence staining of Cerk in the myocardium. Scale bar, 10 μm. **(F)** Quantification of fluorescence intensity of Cerk. Data are mean ± SEM, *n* = 5, *P < 0.05, unpaired two-tailed *t*-test.

Emerging evidence suggests that Cer serves as a biomarker of AMI and also contributes to MI injury (Laaksonen et al., 2016; de Carvalho et al., 2018). However, we found among all the Cers, only Cer 16:0 and Cer 24:1 were significantly higher in the MI group (Figure 3A). Since Cer might convert to Cer1P and the latter contributed highly to the difference of sphingolipid profile between groups (Figures 2C,D), we speculated a conversion of Cer to Cer1P might exist during the reparative phage of MI injury. We next investigated the ratio of Cer1P and Cer. As shown in Figure 4A, all the 13 ratios of Cer1P/Cer except Cer1P 18:2/Cer 18:2 were higher in the MI group compared to the sham group, suggesting an increase of conversion from Cer to Cer1P during the reparative phage.

Considering the critical role of Cerk in controlling both Cer levels and production of Cer1P, we further evaluated the expression of Cerk in heart tissue. As shown in Figures 4B–D, both mRNA level and the protein level of Cerk were significantly higher in the MI group compared to the sham group. Similarly, the expression of Cerk in the border area of heart tissue post-MI was also higher compared to non-infarcted area of heart tissue (Figures 4E,F). Together, these data suggested that upregulated expression of Cerk in the heart after MI might contribute to the conversion of Cer to Cer1P.

## Discussion

Cardiac inflammation and resolution are critical to the severity of cardiac dysfunction post-MI and abnormal lipid metabolism contributes to myocardial injury and remodeling. In the present study, we observed the alternation of the sphingolipid profile during the reparative phage after MI injury. Our results indicated that the sphingolipid profile in heart tissue but not in plasma was largely changed. Meanwhile, we identified several significantly higher or lower levels of metabolites in the heart tissue of the MI group which might serve as biomarkers or contribute to cardiac remodeling. Moreover, we found the ratios of Cer1P to Cer were markedly upregulated in heart after MI, which might be due to the upregulation of the expression of Cerk post-MI.

Abnormal Cer accumulation has been implicated in AMI (de Carvalho et al., 2018; Hadas et al., 2020). Consistently, we also found an increasing trend of the level of myocardial Cer in the reparative phage after MI injury. Because excess Cer was implicated in cardiac lipotoxicity, previous studies focused on the synthesis of Cer and tried to improve heart function by targeting cardiac Cer accumulation. Pharmacologically or genetic inhibition of serine palmitoyltransferase, a rate-limiting enzyme in Cer biosynthesis, reduces fatty acid and prevents cardiac toxicity in a mouse model of dilated cardiomyopathy (Park et al., 2008). Nevertheless, whether the reduction of Cer levels will improve cardiac remodeling is still controversial. Genetic deletion of the serine-palmitoyl transferase long chain 2 (SPTLC2) gene significantly decreased the level of total and individual Cer in the myocardium of mice but did not alleviate acute cardiac dysfunction at 2 weeks post-MI (Ji et al., 2017). Cer, accumulated in the post-ischemic heart, was thought to be mediated by acid sphingomyelinase activity rather than neutral sphingomyelinase activity or *de novo* sphingolipid synthesis (Klevstig et al., 2016). Heterozygote knockout of acid sphingomyelinase (Smpd1) limited Cer accumulation in the post-MI heart without improving cardiac function or survival (Klevstig et al., 2016). Reducing Cer level in mice via the deletion of ceramide synthase 2 (CERS2) leads to the development of progressive myoclonic epilepsy (Mosbech et al., 2014). These failures might be due to that some important downstream metabolites of Cer were simultaneously limited. However, there has been much less emphasis on the downstream metabolites of Cer and the potential effects of Cer1P on cardiac remodeling.

Cer1P has been well characterized to promote proliferation and inhibit apoptosis. The early studies reported that Cer1P induced DNA synthesis in fibroblasts and promoted cellular proliferation and growth (Gomez-Munoz et al., 1995, 1997). Cer1P could also stimulate the proliferation of macrophages (Gangoiti et al., 2010) and C2C12 myoblasts (Gangoiti et al., 2012). In addition, Cer1P attenuated the activation of caspases and prevented DNA fragmentation induced by serum deprivation in macrophages (Gomez-Munoz et al., 2004). Hence, Cer1P serves as a pro-survival player and antagonizes the proapoptotic effects of Cer (Hoeferlin et al., 2013). Moreover, Cer1P inhibited the production of pro-inflammatory cytokines and leukocyte infiltration in pulmonary tissue induced by cigarette smoke in mice, suggesting anti-inflammatory properties of Cer1P (Baudiss et al., 2015). In addition, Cer1P also has a pro-angiogenic role evidenced by stimulating endothelial cell-mediated capillary-like tubule formation *in vitro* and matrigel implant vascularization (Kim et al., 2013). In the present study, we found myocardial Cer1P, including Cer1P 14:0, Cer1P 18:1, Cer1P 18:2, and Cer1P 18:3, were significantly increased at day 7 in the MI group. Besides, Cer1P 18:1 and Cer1P 14:0 contributed highly to the alternation of sphingolipid profile post-MI. Hence, our data suggested that Cer1P might play a vital role during the reparative phase after MI. Nevertheless, whether Cer1P directly involved and exerted an important role in the repair of MI deserves further investigation.

Besides quantifying the levels of metabolites of sphingolipid, we also studied the ratio of Cer1P and Cer. Cer1P is generated by phosphorylation of Cer (N-acyl sphingosine) and is believed to be mainly mediated by Cerk (Boath et al., 2008). The role of Cerk in heart is largely unknown. Cerk was expressed at high level in the heart tissue (Sugiura et al., 2002) and its expression was upregulated in primary cardiomyocytes by treating with the serum of patient with single ventricle congenital heart disease (Garcia et al., 2018). Cerk deficient mice displayed decreased C1P and enhanced Cer, and lacked the capacity to phosphorylate Cer (Graf et al., 2008). However, the genetic deficiency of Cerk improved diet-induced obesity and insulin resistance in mice (Mitsutake et al., 2012), indicating a complicated role of Cer1P. Since Cer1P has diversified roles in the regulation of various physiological and pathological processes, myocardial-specific overexpression of Cerk might reduce local Cer and be beneficial to the reparative process. In further support of this concept is that the overexpression of acid ceramidase (AC), the only enzyme known to hydrolyze Cer and generates sphingosine, could counteract the negative effects of elevated Cer and provide cardioprotection after MI (Hadas et al., 2020). Since gene therapy with adeno-associated virus system has shown promising translational potentials in HF (Eulalio et al., 2012; Gabisonia and Recchia, 2018; Ding et al., 2020), whether cardiac-specifically targeting Cerk has the therapeutic potential in ischemic heart diseases needs further investigation.

Overall, we characterized the significant changes of myocardial sphingolipid profile in the reparative phage after MI injury and observed activation of Cerk/Cer1P pathway. Further work will help to identify the function of the pathway and will lead to a better understanding of the role of sphingolipids in cardiac remodeling after MI.

## Data Availability Statement

The original contributions presented in the study are included in the article/supplementary material, further inquiries can be directed to the corresponding author/s.

## Ethics Statement

The study protocols involving the use of animals were approved by Institutional Animal Care and Use Committee of Tianjin Medical University.

## Author Contributions

TH, QB, and JH designed the research. TH, QB, XH, and WC performed the research and analyzed the data. TH, QB, and JH wrote the manuscript. All authors read and approved the submitted version.

## Conflict of Interest

The authors declare that the research was conducted in the absence of any commercial or financial relationships that could be construed as a potential conflict of interest.

## Footnotes

1 http://www.metaboanalyst.ca
